# Total and free vitamin D status among apparently healthy adults living in Duhok Governorate

**DOI:** 10.1038/s41598-022-05775-x

**Published:** 2022-02-02

**Authors:** Masood Abdulkareem Abdulrahman, Suad Yousif Alkass, Noor Isam Mohammed

**Affiliations:** 1 Department of Public Health, College of Health and Medical Techniques, Duhok Polytechnic University, 61 Zakho Road, 1006 Mazi Qr., P.O.Box 101, Duhok, Kurdistan Region Iraq; 2 Department of Medicinal Chemistry, College of Pharmacy, University of Duhok, Duhok, 1006 AJ Iraq; 3 Department of Medical Laboratory Technology, College of Health and Medical Techniques, Duhok Polytechnic University, Duhok, Iraq

**Keywords:** Biochemistry, Medical research, Risk factors

## Abstract

Serum total 25‐OHD is a main marker of vitamin D which represents the intake and sunlight exposure. Free form of 25‐OHD, the small fraction not bound to a transporter protein has been incorporated as a new marker. This cross-sectional study aimed to evaluate the impact of several factors on total and free vitamin D levels in healthy subjects and to find out if the free form of vitamin D could be a better representative of the body’s vitamin D status. Total and free 25‐OHD were analyzed by ELISA method in a blood sample collected from 391 apparently healthy volunteers (219 female and 172 Male) from Duhok Governorate/Iraq population. Total and free 25‐OHD levels were increased proportionally to BMI with lower values seen in the underweight group, also a significant gender differences in total D3 level with higher values in males (23.90 ± 16.41) ng/ml than females (21.24 ± 15.65) ng/ml was observed. Total and Free 25‐OHD levels were significantly associated with ages, their deficiency most frequent occurs in the younger ages between (16–25) years old. Smokers had higher level of Total 25‐OHD (26.95 ± 19.01) ng/ml and Free 25‐OHD (9.47 ± 4.94) pg/ml than nonsmokers (22.14 ± 14.59) ng/ml and (7.87 ± 4.32) pg/ml respectively. A significant increase in Free 25‐OHD level in the veiled women (9.12 ± 4.64) ng/ml than unveiled (6.16 ± 3.73) ng/ml with a significant positive correlation between Free 25‐OHD level and dress style was also seen. 30% and 33% of the participants whom their daily exposure to sunlight for 30 min and > 1 h respectively were severe deficient in total 25‐OHD. 95% of the participants who had Abnormally low level of free D were exposed for ≥ 30 min to sunlight. Daily exposure to sunlight was negatively associated with Free 25‐OHD level.

## Introduction

Vitamin D, 25-Hydroxy Vitamin D (25-OHD), is a lipophilic hormone that plays essential role in maintenance of calcium and mineralization of bones^1^. To meet biological requirements, 90% of vitamin D (D3) is synthesized endogenously through exposure to sunlight and the rest is exogenously obtained from the diet and fortified foods which could be of two types vitamins D2 (ergocalciferol) and D3(cholecalciferol)^2^. Similarly, both vitamins D3 and D2 undergo first hydroxylation in the liver by the enzyme cytochrome P450 produces 25-hydroxyvitamin D. 25-hydroxy vitamin D3 is the most abundant form of vitamin D in the circulation^3^. Further hydroxylation occurs in the kidney by 1α-hydroxylase enzyme to produce the active form of vitamin D (1, 25(OH)2D)^4^. The nutritional essentiality of vitamin D to maintain good health, also the association between its deficiency with several diseases including hypertension, coronary heart diseases, cardiovascular diseases, cancer, tuberculosis, respiratory tract infection, diabetes and obesity, moreover the improvement after vitamin D administration has been well documented by^5–7^. Logically, this led to an increase the demand for the determination of vitamin D levels and consequently increases the number of scientific papers on this topic in order to prove that its insufficiency or deficiency was the causative factor of that particular disease.

Vitamin D levels can be investigated by the measurement of total 25-OHD (TD) which constituted about 90% of the requested determinations in the laboratory. It refers to both circulating forms (25(OH)D_2_ and D_3_) of the vitamin. The remaining 10% of the requests from metabolites of vitamin D are of 1,25 (OH) 2D which is largely mistakenly requested by the prescriber due to the confusion between both of them^8^. Several causes limited the utilization of 1,25(OH)_2_D as a marker of vitamin D levels due to its short half-life (4–15 h vs 21–30 days of 25-OHD) and because of low concentrations of the final metabolite (picomole vs nanomole), also it might give a false idea of sufficiency owing to the very small amount of 25-OHD that can be converted to it^9^.

To facilitate the work of researchers and clinicians, several new trials have been developed such as measurement of C3 epimer, a stereo‐isomer of 25‐OHD and 24,25-dihydroxy vitamin D (24,25(OH) 2D) a catabolite of 25‐OHD although their clinical importance were not fully understood^10,11^. The measurement of bioavailable and Free 25‐OHD levels have also been incorporated as new markers of vitamin D levels since 1980s but was not used regularly due to lack of its assessment method^12^.

Due to their hydrophobic properties, vitamin D metabolites bind to a protein transporter called vitamin D binding protein (VDBP or DBP). It binds to all the metabolites but has a greater affinity for 25‐OHD, so it binds about 90% of its concentration in blood and the rest 10% binds to albumin with a much lower affinity than VDBP^13^. A small fraction, less than 0.1% of the TD circulates freely, and not bound^14^. The sum of the Free 25‐OHD (FD) and the albumin bound fraction is called the bioavailable 25‐OHD. The investigators believe that the low‐affinity albumin‐25‐OHD complex allows 25‐OHD molecules to be available to exert their biological effects^15^. It is presently unclear whether free or bioavailable 25-OHD is better reflecting the availability of 25-OHD for cells or tissues.

It was concluded previously by^16^ that in a number of clinical situations measuring the free level may provide a better index of vitamin D levels than total levels in such situations. Measurement of FD at the beginning and end of pregnancy gave an optimal monitoring of vitamin D levels^17^. FD gave a statistically significant differences between the infertile versus the control groups whereas the TD couldn’t^18^. The usefulness of FD as a predictor of physical performance in elderly healthy African- American women was also proved by^19^. Elevation of the free form of vitamin D and its association with greater survival was noticed in patients with stages I–III of colorectal cancer^20^.

Intensive searches showed that most of the previous studies evaluated the Free 25‐OHD levels in many diseases and no studies have yet evaluated the free form of vitamin D in the apparently healthy volunteers in Iraq, therefore this cross-sectional study was planned to evaluate the serum TD and FD levels together in the population of Duhok/Kurdistan region/Iraq and to study the association between them. Besides that, the present study has also evaluated the effects of age, gender, smoking, wearing hijab and daily exposure to sunlight on both parameters. Finally, to find out if Free 25‐OHD could be a better representative of the body’s vitamin D levels.

## Subjects and methods

### Subjects and study design

This cross-sectional study was carried out at Central Laboratory, Duhok/Kurdistan Region of Iraq from December to March 2020. A total of 391 apparently healthy volunteers (219 female and 172 Male) with ages ranged between (18 to 70) years old were informed and interviewed about the nature of the study, then were asked to take part in the study and a written consent were obtained from them. A random sampling method was applied to collect the blood samples from the volunteers which were distributed as follows students and academic and administrative staff of two private Universities (Jihan Private University and Nawroz Private University), and Public universities(Duhok Polytechnic University and Duhok University), in addition to employees of some public administrative inside the Duhok governorate. For sample size calculation Cochran’s formula for categorical data was adopted, and this type of sample calculation usually used for large populations.^10^ For more conservative sample size, maximum variability assumed by using an estimated proportion of 0.5. The resultant sample size was 384.6. To allow for non-response or missing data, the sample size was increased by 5%, which added extra 19 individuals so that the final parent sample became 403.

$$ {\text{Sample size }} = \frac{{{\text{Z}}^{{2}} * \, \left( {\text{p}} \right) \, \left( {\text{q}} \right)}}{{{\text{d}}^{{2}} }} $$

where: Z = the desired confidence level (1.96 for 95% confidence level), p = the estimated proportion in the population (0.5), q = is the estimated proportion of an attributed that is present in the population and q = 1 − p, d = the desired level of precision (0.05)

$$ {\text{Sample size}} = \frac{{\left( {{1}.{96}} \right)^{{2}} * 0.{5 }\left( {{ 1} - 0.{5}} \right)}}{{\left( {0.0{5}} \right)^{{2}} }} = {384}.{16 }\left( {{\text{rounded to}} {384}} \right) $$

To allow for non-response, the sample was increased by 5%, 384.16 * 0.05 = 19.2 (rounded to 19), 84 + 19 = 403 it rounded to 400 as final sample size. Later on nine samples excluded due to some technical issues.

The exclusion criteria were those subjects on vitamin D supplements during the last three months, on drug therapy, currently pregnant, chronic and endocrine diseases and any recent infection.

### Ethics statement

This study was formally approved by the Ethics Committee of the Duhok General Directorate of Health, Ministry of Health, under reference number 03122019-8. All study participants signed an informed consent form prior to study procedures, and all methods were performed in accordance to Iraqi guidelines and regulations for biomedical research. No adverse event was reported during this study. The verbal consent was taken from each parent for participation in the study.

### Data collection

A pre-tested questionnaire was submitted for the enrolled cases detailing their ages, genders, marital status, occupation, timing and duration of exposure to sun light, in addition to many other health information, social practices, dietary habits and any medication if present. The participants filled the questionnaires and checked for incomplete answers. Venous blood (5 ml) was withdrawn from selected subjects using disposable syringes. Care was taken to avoid venous stasis during sample collection. The blood was placed in a yellow top tube (serum-separating tube) after removing the needle from the syringe to allow free flow of the blood on the inner wall of the tube to avoid hemolysis during processing and kept at room temperature for 15 min for clotting. The tubes were centrifuged at 4000 rpm for 15 min, and serum samples were then recovered, divided into two parts in Eppendorf tubes, and kept frozen at – 70 °C to be analyzed for TD and FD levels later on. Freeze and thawing cycles of serum samples was avoided. The weight and height were measured while the participants wearing light clothing without shoes, then Body mass index (BMI) was calculated (the ratio between weight (kg) and the squared height (m^2^).

### Assessment of studied variables

The subjects were grouped according to the studied factors as shown below.

BMI according to the National Institutes of Health, the groups division was as follows^21^:

Underweight < 18.5 kg/m^2^, Normal weight = 18.5–24.9 kg/m^2^, Overweight = 25–29.9 kg/m^2^ and Obese ≥ 30 kg/m^2^.

1. Age
  - The participants ages ranged from ≥16 to 70 years were divided into 9 groups (5 years/ group).
2. Smoking
  - Smokers: smoke 1 cigarette/day.
  - Non-smokers: never smoked or quitted smoking 5 years ago.
3. Dress style
  - Wearing Hijab (Veiled): Covers their head.
  - Not wearing hijab (unveiled).
4. Daily exposure to sunlight:
  - 15 min.
  - 20 min.
  - 30 min.
  - (30–60) min.
  - > 60 min.

### Biochemical analysis

The TD and FD levels were assayed using enzyme-linked immunosorbent assay (ELISA) technique. The assessment was performed by using Elisa Reader/Microplate Reader (WHY101T)/Rayta/China. TD level was measured using ELISA kit supplied by Monobind Inc./USA, product code (9425-300). Free D3 was measured using ELISA kit supplied by DIA source ImmunoAssays/Belgium, product code 3L-FVD-04 rev. B (10/17). The assessment was based on a two-step immunoassay procedure.

### Determination of vitamin D levels

Vitamin D levels is categorized according to Endocrine Society Guidelines as deficiency, insufficiency, and sufficiency based on serum TD level below 20 ng/ml (50 nmol/L), 21–29 ng/ml (52.5–72.5 nmol/L), and 30–100 ng/ml (75–250 nmol/L), respectively. A cutoff of < 10 ng/ml (< 25 nmol/L) is considered to determine severe vitamin D deficiency^22^.

### Statistical analysis

Various quantitative data analyses were used, starting with the descriptive analysis where all variables were expressed as mean ± standard deviation. The covariates were considered as confounding factors included Gender, Age, Body Mass Index, Exposure to Sunlight, Tobacco Smoking and Wearing Hijab, and the relationships between these factors with TD and FD levels were also investigated through clustered bar charts. Student’s t-test was used to determine if there is a significant difference between groups of TD and FD, and Chi-square test was also used to assess the relationships between categorical variables, such as Gender with TD, Age groups with Gender, Smoking with FD, Gender with FD, and Smoking with TD. As almost all the variables in our study are categorical, Spearman’s correlations coefficients were used to analyze the association between TD and FD levels with all other variables (factors). The value of P < 0.05 were considered as a statistically significant for all the statistical analysis conducted in this study. All data were analyzed by the SPSS (IBM Corporation, New York, NY, USA) statistical package (Version 25.0).

### Ethical approval

The study was approved by the Committee of Research Ethics in the Directorate General of Health in Duhok province. The verbal consent was taken from each parent for participation in the study.

## Results

Out of 391 subjects recruited in this study, 219(56%) were females and 172(44%) were males. When the subjects were divided based on ages in this study, a non-significant variation on the mean levels of TD and FD was observed. The main age groups were 26–30 years (16.9%) and 31–35 years (16.9%), while the smallest group was 51–55 years (3.3%) as shown in Table 1. (24%) of the participants had severe TD which is more common among individuals with ages ranged between (26–30) years old, whereas (32%) of the involved subjects are T25-OHD deficient which is most frequent within (31–35) and (36–40) years old groups. The subjects who had sufficient T25-OHD constituted about (24%) of the total participants is more prevalent in (31–35) years old group. More than 50% of the participant had their TD levels less than 20 ng/ml specifically in the ages between (16–40) years old. The results of FD according to different ages indicated that 5% of the subjects had a non-significant low value which is most frequent occurs in the younger ages between (16–25) years old Table 2. Furthermore, a significant positive association was shown between age and both vitamin D forms levels, Table 10. The mean values of total and free forms were calculated at different age groups, the least TD level was noticed in the youngest groups (16–25) years and increased gradually with maximum level seen in the older group (≥ 56) years old. The results of this study demonstrated a significant gender differences of TD level with a higher mean value in males (23.90 ± 16.41) ng/ml than females (21.24 ± 15.65) ng/ml as shown in Table 3. Older males ≥ 26 years showed slightly higher TD levels than females with in the same age group with the older age group give higher level, they had sufficient vitamin D level. Regarding FD level, also the highest level noticed was among males (10.67 ± 2.54) pg/ml in (> 56) years old and among females in (51–55) years age group (10.90 ± 4.09) pg/ml. Furthermore, FD level was increased gradually and proportionally to increasing ages Table 1.

**Table 1:** Descriptive statistics with mean and standard deviation of Total 25-OHD and Free 25-OHD in respect to age groups and gender.

Age groups /years	Gender						Total		
	Male			Female					
	No. (%)	TD (ng/ml) Mean ± SD	FD (pg/ml) Mean ± SD	No. (%)	TD (ng/ml) Mean ± SD	FD (pg/ml) Mean ± SD	No. (%)	TD (ng/ml) Mean ± SD	FD (pg/ml) Mean ± SD
16–20	14 (3.6)	19.01 ± 9.16	3.97 ± 1.48	36 (9.2)	19.58 ± 15.16	5.01 ± 3.42	50 (12.8)	19.41 ± 13.75	4.72 ± 3.04
21–25	13 (3.3)	17.02 ± 7.82	5.75 ± 3.61	34 (8.7)	17.99 ± 14.43	5.97 ± 4.12	47 (12.0)	17.72 ± 12.95	5.91 ± 3.98
26–30	38 (9.7)	23.52 ± 18.44	8.44 ± 4.29	28 (7.2)	18.19 ± 12.84	8.44 ± 4.00	66 (16.9)	21.26 ± 16.51	8.44 ± 4.17
31–35	32 (8.2)	23.17 ± 12.59	9.42 ± 6.02	34 (8.7)	26.17 ± 19.69	9.25 ± 4.03	66 (16.9)	24.72 ± 16.70	9.33 ± 5.09
36–40	30 (7.7)	27.70 ± 13.97	9.48 ± 4.52	30 (7.7)	26.66 ± 16.78	8.26 ± 4.19	60 (15.3)	26.68 ± 15.47	8.87 ± 4.40
41–45	16 (4.1)	23.22 ± 17.52	8.48 ± 3.97	23 (5.9)	19.91 ± 14.34	8.34 ± 4.60	39 (10.0)	21.26 ± 15.81	8.40 ± 4.35
46–50	12 (3.1)	20.65 ± 13.27	9.31 ± 2.55	19 (4.9)	18.37 ± 13.06	9.78 ± 7.03	31 (7.9)	18.25 ± 13.19	9.59 ± 5.73
51–55	6 (1.5)	20.13 ± 12.05	8.61 ± 3.08	7 (1.8)	23.68 ± 7.03	10.90 ± 3.79	13 (3.3)	22.04 ± 9.81	9.84 ± 3.66
≥ 56	11 (2.8)	34.94 ± 28.34	10.76 ± 2.52	8 (2.0)	24.08 ± 12.75	7.17 ± 4.04	19 (4.9)	32.10 ± 24.75	9.25 ± 3.80
Total	172 (44.0)	23.90 ± 16.41	8.46 ± 4.60	219 (56.0)	21.24 ± 15.65	7.73 ± 4.69	391 (100.0)	24.41 ± 16.05	8.05 ± 4.66

(P-value = 0.297).

**Table 2:** Total 25-OHD and Free 25-OHD levels in respect to age groups.

Age groups/years	TD (ng/ml)				FD (pg/ml)		Total
	< 10 severe deficient no. (%)	10–20 deficient no. (%)	20–29 insufficient no. (%)	≥ 30 sufficient no. (%)	2.5–35 normal no. (%)	< 2.5 deficient no. (%)	
16–20	16 (4.1)	14 (3.6)	10 (2.6)	10 (2.6)	46 (11.8)	4 (1.0)	50 (12.8)
21–25	15 (3.8)	18 (4.6)	7 (1.8)	7 (1.8)	43 (11.0)	4 (1.0)	47 (12.0)
26–30	19 (4.9)	19 (4.9)	14 (3.6)	14 (3.6)	63 (16.1)	3 (0.8)	66 (16.9)
31–35	12 (3.1)	21 (5.4)	13 (3.3)	20 (5.1)	64 (16.4)	2 (0.5)	66 (16.9)
36–40	6 (1.5)	22 (5.6)	15 (3.8)	17 (4.3)	57 (14.6)	3 (0.8)	60 (15.3)
41–45	13 (3.3)	10 (2.6)	7 (1.8)	9 (2.3)	36 (9.2)	3 (0.8)	39 (10.0)
46–50	8 (2.0)	11 (2.8)	7 (1.8)	5 (1.3)	28 (7.2)	3 (0.8)	31 (7.9)
51–55	1 (0.3)	6 (1.5)	2 (0.5)	9 (2.3)	13 (3.3)	0 (0.0)	13 (3.3)
56 and above	4 (1.0)	4 (1.0)	2 (0.5)	9 (2.3)	18 (4.6)	1 (0.3)	19 (4.9)
Total	94 (24.0)	125 (32.0)	77 (19.7)	95 (24.3)	368 (94.1)	23 (5.9)	391 (100.0)

P-value of TD = 0.297, P-value of FD = 0.846.

**Table 3:** Mean and standard deviation of Total 25-OHD level in respect to gender.

TD (ng/ml)	Gender				Total	
	Male		Female			
	No. (%)	TD (ng/ml) Mean ± SD	No. (%)	TD (ng/ml) Mean ± SD	No. (%)	TD (ng/ml) Mean ± SD
< 10 severe deficient	28 (7.2)	7.80 ± 1.72	66 (16.9)	6.07 ± 2.16	94 (24.0)	6.59 ± 2.18
10–20 deficient	63 (16.1)	15.36 ± 3.22	62 (15.9)	15.43 ± 3.40	125 (32.0)	15.39 ± 3.31
21–29 insufficient	39 (10.0)	24.29 ± 2.52	38 (7.9)	25.05 ± 2.59	77 (19.7)	24.67 ± 2.58
≥ 30 sufficient	42 (10.7)	47.09 ± 16.12	53 (13.6)	44.18 ± 11.20	95 (24.3)	45.46 ± 13.67
Total	172 (44.0)	23.90 ± 16.41	219 (56.0)	21.24 ± 15.65	391 (100.0)	24.41 ± 16.05

*P*-value < 0.011.

**Figure 1:**
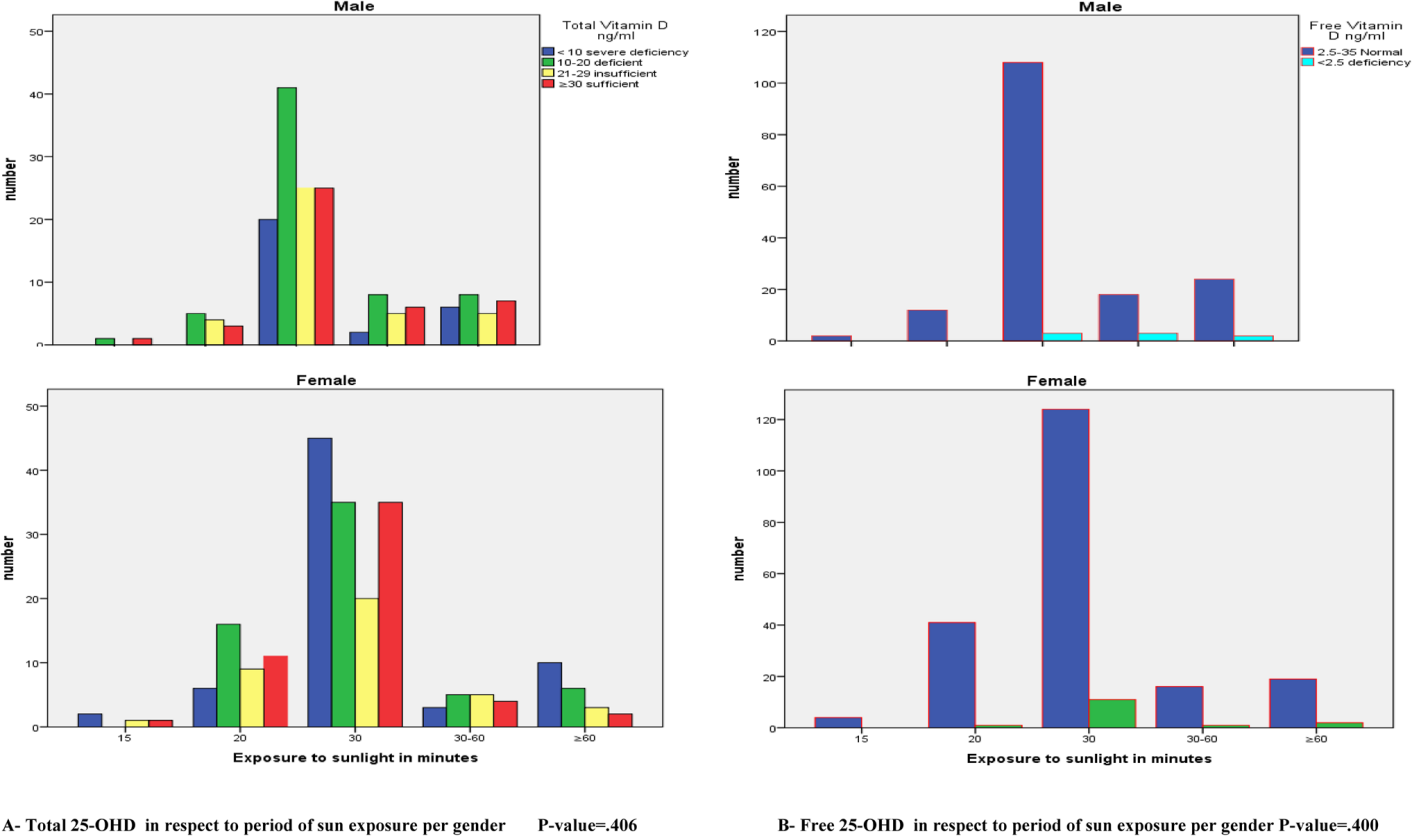
Total and Free 25-OHD in respect to period of sun exposure per gender. (**A**) Total 25-OHD in respect to period of sun exposure per gender P-value = 0.406. (**B**) Free 25-OHD in respect to period of sun exposure per gender P-value = 0.400.

About 53% of males and 58% of females who participated in this study were suffering from vitamin D deficiency. The severity of vitamin D deficiency is more common in females 17% than males (7%) of the whole participants. Regarding free vitamin D level, (95%) of the participants had normal FD level and a non-significant effect of gender was noticed with a slightly larger values in males than females, Tables 3 and 4 and Fig. 1 for TD and FD results, respectively.

**Table 4:** Mean and standard deviation of Free 25-OHD level in respect to gender.

FD (pg/ml)	Gender				Total	
	Male		Female			
	No. (%)	FD (pg/ml) Mean ± SD	No. (%)	FD (pg/ml) Mean ± SD	No. (%)	FD (pg/ml) Mean ± SD
2.5–35 normal	164 (41.9)	8.79 ± 4.44	204 (52.2)	8.17 ± 4.55	368 (94.1)	8.45 ± 4.51
< 2.5 deficiency	8 (2.0)	1.60 ± 0.32	15 (3.8)	1.72 ± 0.48	23 (5.9)	1.68 ± 0.44
Total	172 (44.0)	8.46 ± 4.60	219 (156.0)	7.73 ± 4.69	391 (100.0)	8.05 ± 4.66

P-value = 0.395.

A non-significant increase in TD with an increase in BMI was observed, males had higher levels of vitamin D as compared to the females with in the same group. The highest mean level of TD3 among males in obese group (29.62 ± 24.67) and among females in overweight group (22.47 ± 17.62). Anon significant increase in FD3 level proportionally to BMI was clearly resulted with the lowest level seen in the underweight subjects (5.88 ± 4.40). The values of TD (11.58 ng/ml) and FD (and 6.32 pg/ml) were lower in underweight subjects in comparison to obese subjects (TD 23.35 ng/ml and FD 8.51 pg/ml). Their levels in underweight individuals were the lowest values found while the highest values were found for obese subjects (Tables 5 and 6).

**Table 5:** Mean and standard deviation of Total 25-OHD level in respect to BMI.

BMI (Kg/m^2)^	Gender				Total	
	Male		Female			
	No. (%)	TD (ng/ml) Mean ± SD	No. (%)	TD (ng/ml) Mean ± SD	No. (%)	TD (ng/ml) Mean ± SD
Underweight < 18.5	2 (0.5)	12.74 ± 2.49	9 (2.3)	11.32 ± 7.95	11 (2.8)	11.58 ± 7.29
Normal 18.5–24.9	78 (19.9)	23.00 ± 15.24	98 (25.1)	21.65 ± 15.52	176 (45.0)	22.25 ± 15.41
Overweight 25–29.9	70 (17.9)	23.42 ± 14.03	57 (14.6)	22.47 ± 17.62	127 (32.5)	22.99 ± 15.75
Obese ≥ 30	22 (5.6)	29.62 ± 24.67	55 (14.1)	20.84 ± 13.99	77 (19.7)	23.35 ± 18.15
Total	172 (44.0)	23.90 ± 16.41	219 (56.0)	21.24 ± 15.65	391 (100.0)	22.41 ± 16.05

P-value = 0.620.

**Table 6:** Mean and standard deviation of Free 25-OHD level in respect to BMI.

BMI (Kg/m^2^)	Gender				Total	
	Male		Female			
	No. (%)	FD (pg/ml) Mean ± SD	No. (%)	FD (pg/ml) Mean ± SD	No. (%)	FD (pg/ml) Mean ± SD
Underweight < 18.5	2 (0.5)	8.33 ± 4.30	9 (2.3)	5.88 ± 4.40	11 (2.8)	6.32 ± 4.48
Normal 18.5–24.9	78 (19.9)	8.39 ± 5.04	98 (25.1)	7.10 ± 4.25	176 (45.0)	7.67 ± 4.66
Overweight 25–29.9	70 (17.9)	8.15 ± 4.36	57 (14.6)	8.79 ± 4.77	127 (32.5)	8.44 ± 4.56
Obese ≥ 30	22 (5.6)	8.65 ± 3.34	55 (14.1)	8.06 ± 5.11	77 (19.7)	8.51 ± 4.72
Total	172 (44.0)	8.46 ± 4.60	219 (56.0)	7.73 ± 4.69	391 (100.0)	8.05 ± 4.60

**Figure 2:**
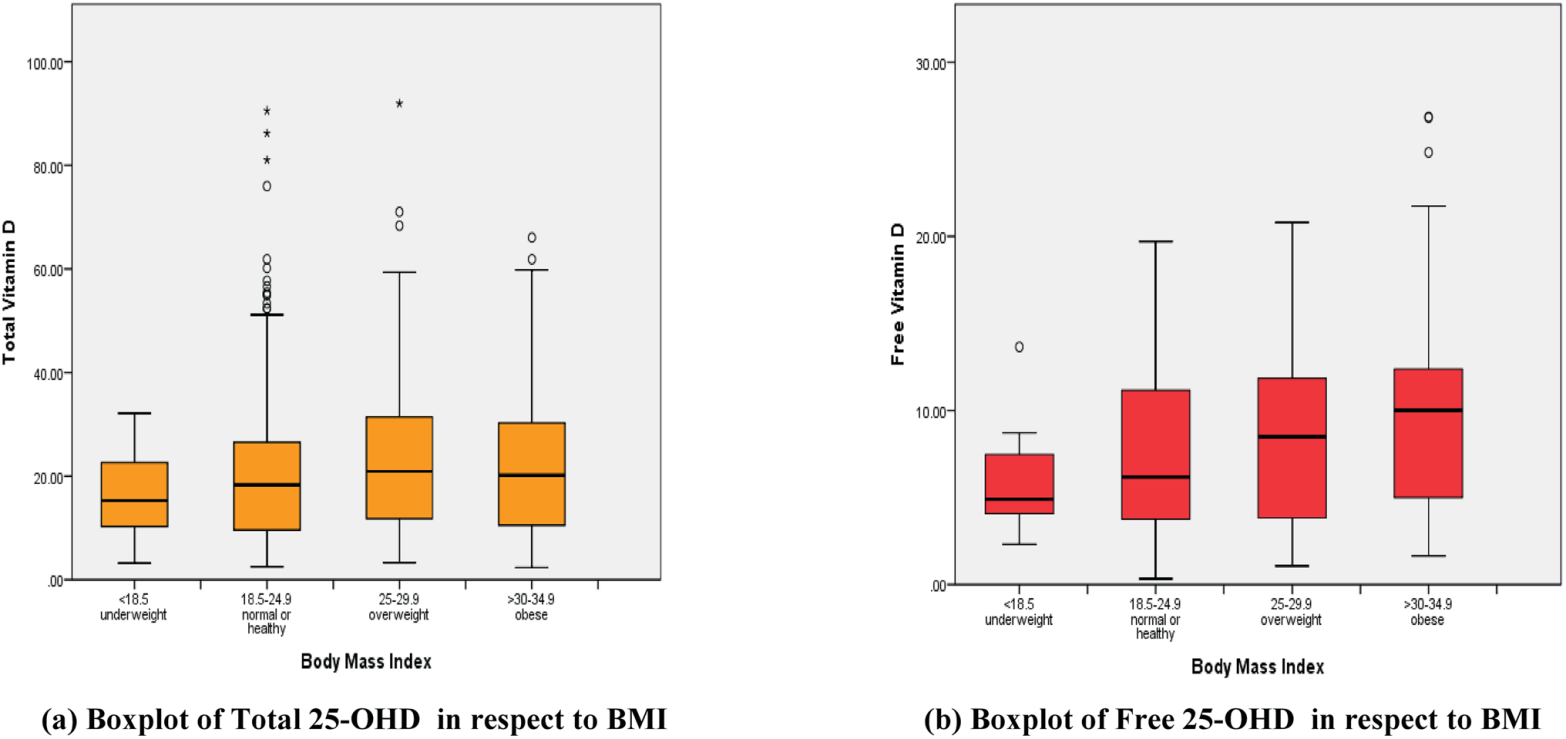
(**a**) Boxplot of Total 25-OHD in respect to BMI, (**b**) Boxplot of Free 25-OHD in respect to BMI.

**Figure 3:**
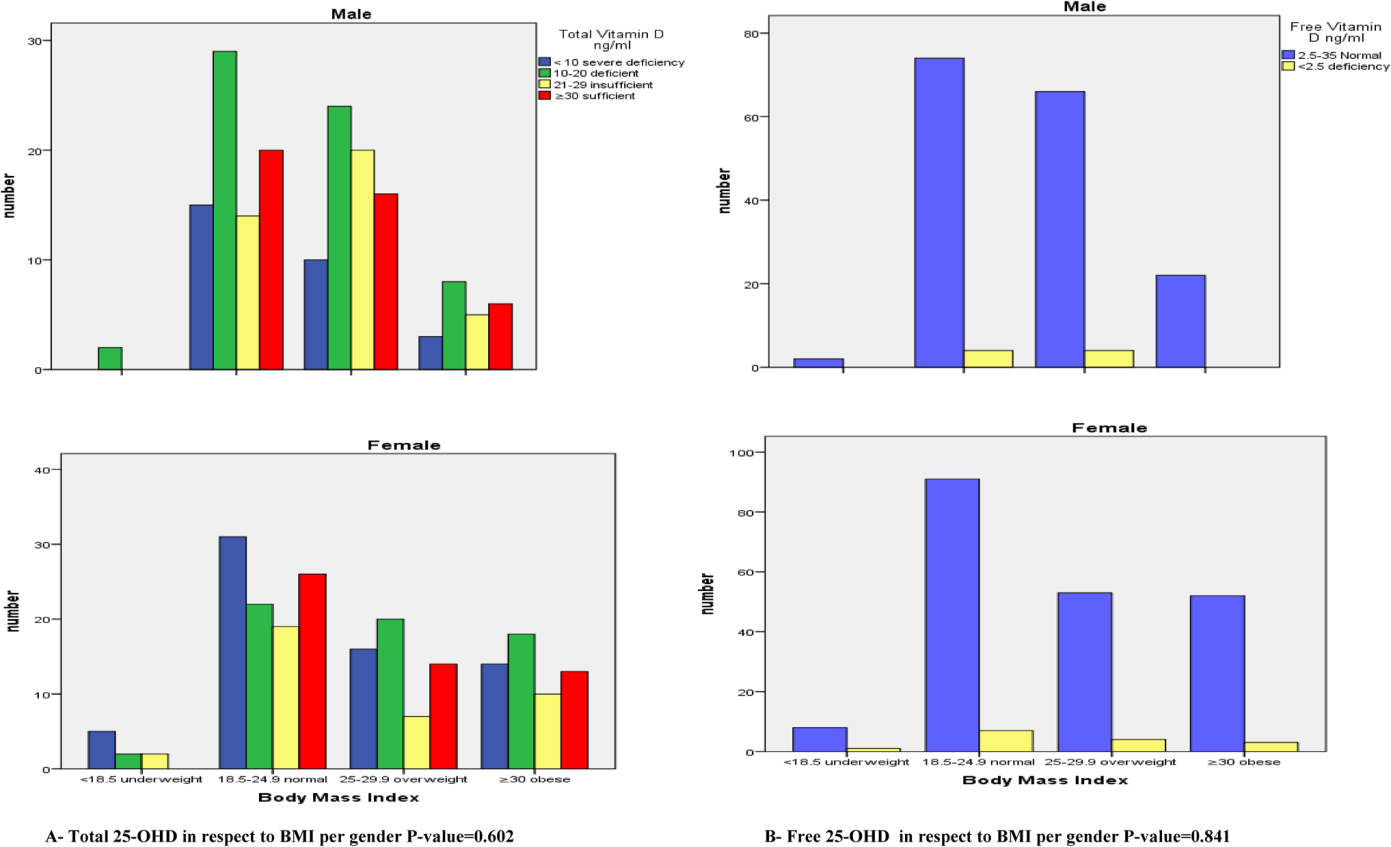
Total and Free 25-OHD in respect to BMI per gender. (**A**) Total 25-OHD in respect to BMI per gender P-value = 0.602, (**B**) Free 25-OHD in respect to BMI per gender P-value = 0.841.

BMI was positively associated with FD level. On looking at TD and FD levels according to BMI per gender as shown in Figs. 2 and 3, severe deficiency of TD levels is most common in the under and normal weight females whereas in the overweight and obese females most of them were deficient in TD level. Regarding the males results, the deficiency was most common in all the groups. Regarding FD levels, the abnormally low levels of it was prevalent in the normal weight subjects. Most of the obese males and females showed normal levels of FD.

**Figure 4:**
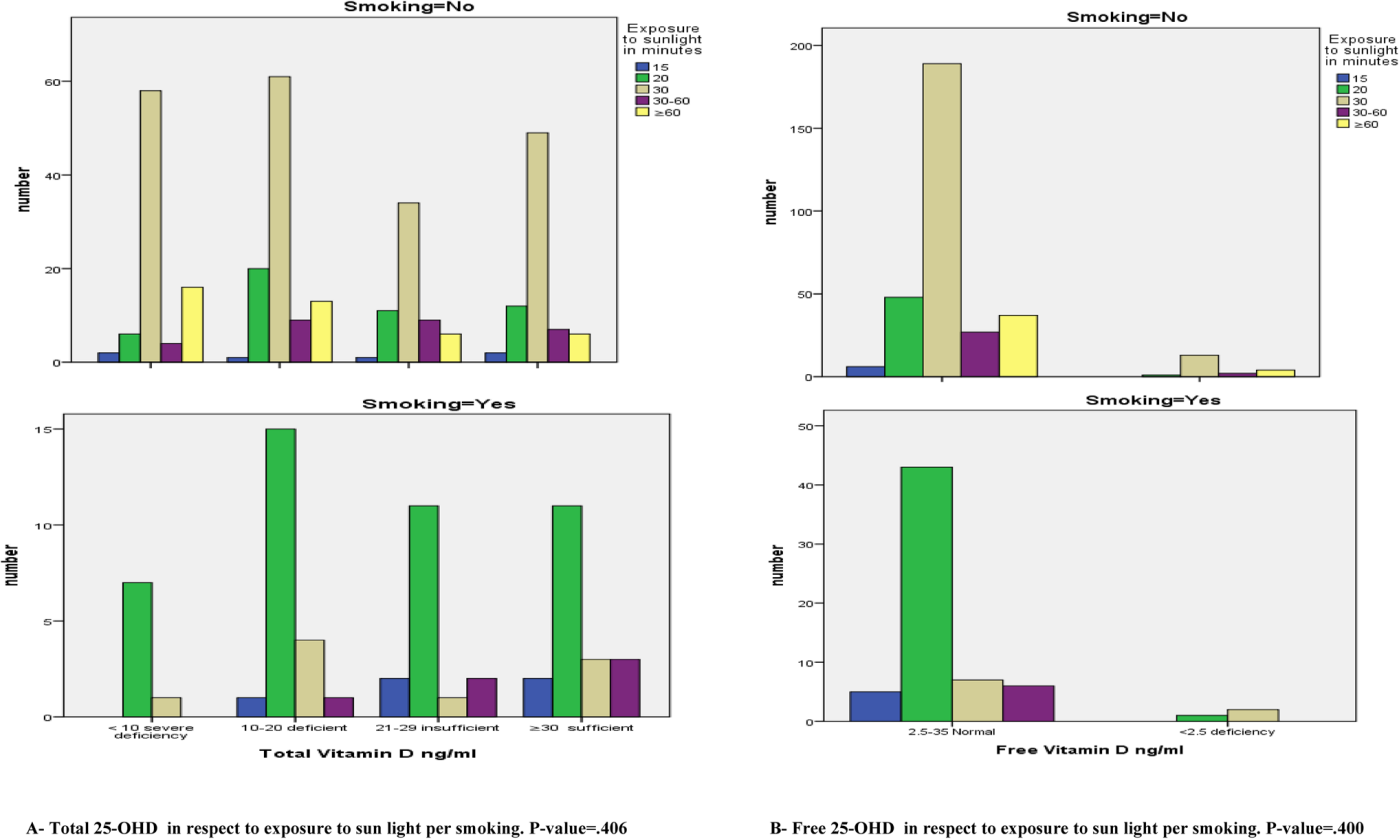
Total and Free 25-OHD in respect to exposure to sun light per smoking. (**A**) Total 25-OHD in respect to exposure to sun light per smoking. P-value = 0.406, (**B**) Free 25-OHD in respect to exposure to sun light per smoking. P-value = 0.400.

**Figure 5:**
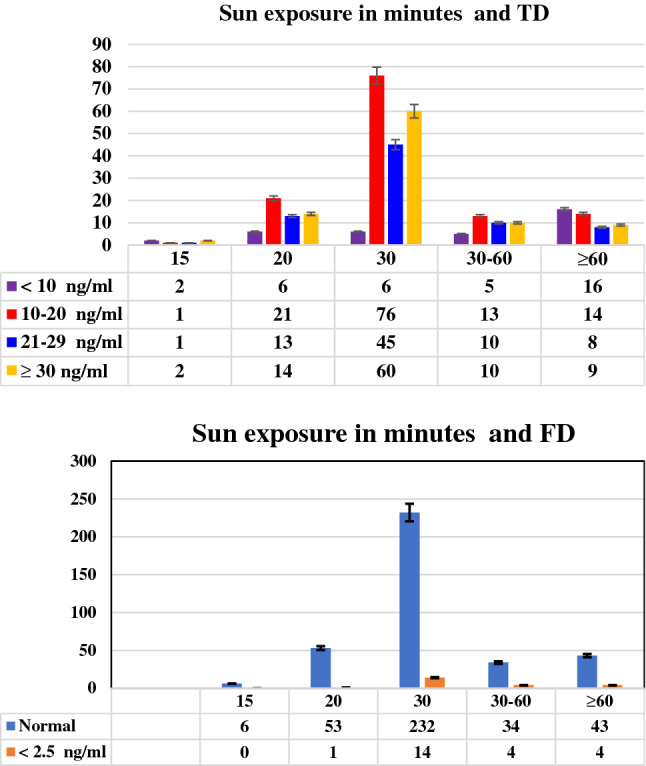
Total and Free 25-OHD in respect to exposure to sun light.

Smokers had a higher but non-significant level of TD (26.95 ± 19.01) ng/ml and FD (9.47 ± 4.94) pg/ml than nonsmokers (22.14 ± 14.59) ng/ml and (7.87 ± 4.32) pg/ml respectively. Moreover, a positive association was found between smoking with both TD and FD levels, Table 7. Smoking effect on TD and FD levels per exposure to sunlight demonstrated that the majority of the smokers were exposed for 20 min to Ultraviolet (UV) light and most of them were deficient in TD followed by the insufficient and sufficient groups shown in Figs. 4 and 5. The severe deficient group is the least one in smokers whom their exposure to UV light for ≥ 20 min. The majority of smokers had normal level of FD level. The abnormally low results found in FD were in the smokers exposed for 20 and 30 min to UV light.

**Table 7:** Mean and standard deviation of Total 25-OHD and F25-OHD in respect to smoking.

Smoking status	Male			Female			Total		
	No (%)	TD (ng/ml) Mean ± SD	FD (pg/ml) Mean ± SD	No (%)	TD (ng/ml) Mean ± SD	FD (pg/ml) Mean ± SD	No (%)	TD* (ng/ml) Mean ± SD	FD** (pg/ml) Mean ± SD
Smokers	63 (16.1)	26.95 ± 19.01	9.47 ± 4.94	1 (1.6)	31.78 ± NA	5.10 ± NA	64 (16.4)	27.02 ± 18.87	9.40 ± 4.93
Non smokers	109 (27.9)	22.14 ± 14.59	7.87 ± 4.32	218 (55.8)	21.17 ± 15.71	7.74 ± 4.71	327 (83.6)	21.50 ± 15.33	7.78 ± 4.58
Total	172 (44.0)	23.90 ± 16.41	8.46 ± 4.60	219 (56.0)	21.24 ± 15.65	7.73 ± 4.69	391 (100.0)	24.41 ± 16.05	8.05 ± 4.66

*P-value = 0.099, **P-value = 0.656, *NA* Not applicable.

Regarding the effect of dress style, the prevalence of severe deficiency, deficiency, insufficiency and sufficient vitamin D level was higher in the covered group than the uncovered women were detected, it was expected result since the number of the covered women were doubled the uncovered women involved in this study. Also, the mean serum level of TD in the veiled women was non-significantly higher than the unveiled women with in the same group, Table 8.

**Table 8:** Mean and standard deviation of Total 25-OHD level in respect to dress style.

TD (ng/ml)	Wearing Hijab				Total	
	Yes		No			
	No (%)	TD Mean ± SD	No (%)	TD Mean ± SD	No (%)	TD Mean ± SD
< 10 severe deficiency	43 (19.6)	6.01 ± 2.30	23 (10.5)	6.17 ± 1.96	66 (30.1)	6.07 ± 2.17
10–20 deficient	40 (18.3)	16.02 ± 3.34	22 (10.0)	14.37 ± 3.39	62 (28.3)	15.43 ± 3.42
21–29 insufficient	27 (12.3)	25.08 ± 2.79	11 (5.0)	24.98 ± 2.28	38 (17.4)	25.05 ± 2.62
≥ 30 sufficient	38 (17.4)	45.67 ± 11.94	15 (6.8)	40.69 ± 8.49	53 (24.2)	44.26 ± 11.23
Total	148 (67.6)	22.38 ± 16.51	71 (32.4)	18.92 ± 13.68	219 (100.0)	21.26 ± 15.70

P-value = 0.786.

As there are no previous studies on the impact of the dress style of females on Free vitamin D level, the current result could be considered as the first one in this respect. There is a significant increase in FD3 level in the veiled women (9.0.12 ± 4.64) ng/ml as compared to unveiled (6.0.16 ± 3.73) ng/ml Table 9. Furthermore, there is a significant positive correlation between FD level and dress style whereas a nonsignificant correlation was found with TD level.

**Table 9:** Mean and standard deviation of Free 25-OHD level in respect to dress style.

FD (pg/ml)	Wearing Hijab				Total	
	Yes		No			
	No (%)	FD Mean ± SD	No (%)	FD Mean ± SD	No (%)	FD Mean ± SD
2.4–35 normal	138 (63.0)	9.12 ± 4.64	66 (30.1)	6.16 ± 3.73	204 (93.2)	8.16 ± 4.57
< 2.4 deficiency	10 (4.6)	1.84 ± 0.36	5 (2.3)	1.47 ± 0.68	15 (6.8)	1.72 ± 0.50
Total	148 (67.6)	8.63 ± 4.84	71 (32.4)	5.83 ± 3.80	219 (100.0)	7.72 ± 4.71

P-value = 0.006.

The results of the present work demonstrated that 63% of the subjects, their daily exposure to sunlight was 30 min, yet they weren’t protected from becoming TD deficient. 30% and 26% of them they were deficient and severe deficient respectively. Also, within a group whom their exposure to sunlight was for 30 min/day, the majority of them constituted (69%, 61%) within their subgroups had severe deficient and deficient in TD respectively. Strangely, 33% of the participants in the group of exposure for > 1 h to sunlight, they were suffered from severe TD deficiency. The majority of the females whom exposed for 30 and ≥ 60 were severe deficient in TD levels whereas the majority of males were deficient.

Regarding FD results versus daily exposure to sunlight, just 6% of the participants had abnormally low results of FD and most of them were females their exposure to sunlight was for 30 min/day and 8 of the rest they exposed for more than 1 h to sunlight (Figs. 4 and 5). Using the Spearman’s correlation coefficient (r) to study how the TD and FD affected by different factors (Table 10), the results shows that the total was correlated to the ages and smoking factors whereas the free form was correlated to the ages, BMI, smoking, dress style and the period of exposure to sunlight.

**Table 10:** Spearman’s correlations relations between Total 25-OHD and Free 25-OHD with different variables.

Variables	Gender	Age	BMI	Smoking	Wearing Hijab	Exposure to sunlight
**TD**						
r**	− 0.095	0.111*	0.046	0.114	− 0.039	− 0.063
P-value*	0.059	0.029	0.366	0.024	0.447	0.216
**FD**						
r**	0.046	− 0.020	− 0.035	− 0.022	0.030	0.097
P-value*	0.360	0.693	0.487	0.658	0.549	0.056

*Correlation is significant at ≤ 0.05 level (2-tailed).

**Spearman Correlation.

On comparison between TD and FD levels and how the subjects were distributed according to their levels (Table 11), our data indicated that among the deficient subjects (TD level < 20 ng/ml) who participated in this study (219), 3% of them showed abnormal FD level. On the other hand, among the 95 subjects whom their TD levels were sufficient (> 30 ng/ml), 2% of them showed abnormal level of FD. It can be concluded here that FD cannot detect all the deficient individuals in TD and the individuals with sufficient TD may be were deficient in FD. So, measurement of FD level in healthy individuals doesn’t give the real vitamin D levels in healthy individuals and it wasn’t a better representative of the real vitamin D levels.

**Table 11:** Comparison between Total 25-OHD versus Free 25-OHD.

TD (ng/ml)	FD pg/ml		Total
	Normal (2.5–35) No. (%)	< 2.5 deficient No. (%)	
< 10 severe deficiency	87 (22.3)	7 (1.8)	94 (24.0)
10–20 deficient	121 (30.9)	4 (1.0)	125 (32.0)
21–29 insufficient	74 (18.9)	3 (0.8)	77 (19.7)
≥ 30 sufficient	86 (22.0)	9 (2.3)	95 (24.3)
Total	368 (94.1)	23 (5.9)	391 (100.0)

P-value = 0.187.

## Discussion

The results of this study demonstrated a significant gender differences of vitamin D level with a higher mean value in males (23.90 ± 16.41) ng/ml than females (21.24 ± 15.65) ng/ml. About 53% of males and 58% of females who were participated in this study were suffering from vitamin D deficiency. The severity of vitamin D deficiency is more common in females 17% than males (7%) of the whole participants. Regarding Free vitamin D level, (95%) of the participants had normal FD level and a non-significant effect of gender was noticed with a slightly larger values males than females.

Lower 25(OH) D levels were also observed in females, as compared to males in^23,24^. Similarly, many other previous studies were done in different Governorate of Iraq. A total of 3520 subjects were enrolled in a study done in central Laboratory in Suleimani, consisting of 574 men and 2946 women, it showed a significant difference between the mean values of vitamin D level in males (16.23 ± 12.86) which is higher than females (13.32 ± 11.6) ng/ml^25^. Another group of volunteers were tested for their vitamin D levels at Kufa Governorate, the results indicated a significant higher level of vitamin D in males (24.56 ± 5.52) as compared to the females (14.37 ± 1086) ng/ml^26^.

The mean serum level of vitamin D in 120 postmenopausal women studied at Karbala Governorate was 13.5 ng/ml, whereas a child bearing group of 100 women was 17.3 ng/ml. The males in the same study showed higher mean level of vitamin D 18.5 ng/ml for the young ages and 14.6 ng/ml for elderly^27^.

Another study was done in Erbil city with the involvement of 10,823 subjects of different ages and both genders. 75% of the participants where females, the mean value of vitamin D level in females was (14.67 ± 22.14) ng/ml which is lower than its corresponding level in males (15.05 ± 16.88) ng/ml^28^.

After a revision of all of the above-mentioned studies done in different Governorates of Iraq and comparing it to the results of the current study, all studies agreed that there are a significant gender differences of vitamin D level and the males had higher mean levels of vitamin D than females. Interestingly, the data of this study showed better vitamin D levels of both genders compared to other Governorates, besides that, the results of vitamin D in females where very close to that of males and much higher than the females studied in other Governorates of Iraq. The outdoor activity of males in addition to excess adipose tissue of females compared with males were suggested as a causal factor of gender variations^29^.

Gender-associated variations in 25-OH Vitamin D levels also could be related to androgen-associated changes in the concentration of Vitamin D-binding protein, precursor production by skin, and 25-hydroxilation in the liver^30^. Moreover, it probably reflects the cultural and religious practices leading to less skin exposure in women than in men^31^.

When the subjects were divided based on ages in this study, a non-significant variation on the mean levels of TD and FD was observed. Vitamin D deficiency is more prevalent in younger ages whereas older ages specifically more than 50 years old, about 50% of the involved subjects in this group they had sufficient vitamin D level and the rest had either insufficient or deficient vitamin D level. Similarly, the results of FD according to different ages indicated that 5% of the subjects had a non-significant low value which is most frequently occurs in the younger ages between (16–25) years old. Furthermore, a significant positive association was shown between age and both TD and FD levels. Bischof and co-workers in 2006 also found similar association of ages with 25-OH-D.

When the subjects in Sulaymaniyah city/Iraq were grouped according to their ages into five groups (15 years/group), Abdullah and his collaborators in 2018 found that the mean vitamin D level increased gradually from 8.6 ng/ml in the first and younger subjects (16–30) years to 14.8 ng/ml in older (> 80) years. Their explanation was that elders may have more healthy foods and their exposure to sunlight more frequently than younger^25^. Another research group, in their study which was carried out in Iran indicated that serum level of 25-hydroxyvitamin D did not decline with age, their finding was considered very similar to this work^32^.

To confirm the above findings, the mean values of TD and FD were calculated at different age groups, the least TD level was noticed in the youngest groups (16–25) years old then increased gradually with maximum level seen in the older group (> 56) years old. The males showed slightly higher TD3 levels than females with in the same age group with the older age group give higher level, they had sufficient vitamin D level. Regarding FD level, also the highest level noticed was among males (10.67 ± 2.54) pg/ml in (> 56) years old and among females in (51–55) years age group (10.90 ± 4.09) pg/ml. Furthermore, FD level was increased gradually and proportionally to increasing ages. Its worthy to mention here that this is the first-time aging effect on the level of TD and specifically FD was studied in details and this result could be considered as the first one in this aspect.

However, our results agree with the above-mentioned studies but disagree with a previous study which was demonstrated that aging affect vitamin D level through different aspects, among them, decreased calcium absorption, intestinal resistance to circulating 1,25(OH)_2_D, decreased vitamin D receptors, decreased production of 1,25(OH)_2_D by the aging kidney, decreased skin production of vitamin D and deficiency of the substrate to produce vitamin D^33^.

A non-significant increase in TD and FD with an increase in BMI was observed, males had higher levels of vitamin D as compared to the females with in the same group also was indicated. The highest mean level of TD among males in obese group (29.62 ± 24.67) and among females in overweight group (22.47 ± 17.62). Similar results were also found in FD according to BMI specifically in females, anon significant increase in its level proportionally to BMI was clearly resulted with the lowest level seen in the underweight subjects (5.88 ± 4.40). When the results of BMI were drawn per gender according to TD levels, it revealed that the females were more prone to severe deficiency, especially in the normal weight females. The prevalence of severe cases was decreased with increasing the BMI. Likewise, the prevalence of severe cases in males declined with increasing BMI, but the majority of them were mostly prone to deficiency in TD levels.

Whether the storage of vitamin D and 25(OH)D in adipose and other tissues is of clinical importance is uncertain and previous studies found that the stored amount has only negligible effects on serum 25(OH)D levels^34,35^. It was reasonable that vitamin D metabolites are gradually released from adipose or other tissues into the circulatory system to prevents serum 25(OH)D from falling to critically low levels during the winter^36^. It’s very important to mention here that neither BMI nor body weight levels reflect the percentage of body fat. To clarify this point, athletes’ persons may have high BMI and may be considered overweight or obese while they have low total fat mass^37^.

It was expected that obese persons have the tendency to produce more vitamin D in the skin than normal weight persons since they have larger body surface area with the same amount of provitamin D (7-dehydrocholesterol) per unit body surface area^38^. Its worthy to mention that regardless of the increase in TD level occurred with the increase in BMI, the result found was not significant. A similar result was obtained in a prospective study conducted in Korea with the involvement of 1080 subjects, besides another local study at Kufa University/Iraq involving 273 individuals. Both mentioned studies revealed a non-significant association between BMI and serum levels of vitamin D^39,40^.

The results regarding vitamin D levels in smokers of this study were unexpected, Smokers had a higher but non-significant level of TD (26.95 ± 19.01) ng/ml and FD (9.47 ± 4.94) pg/ml than nonsmokers (22.14 ± 14.59) ng/ml and (7.87 ± 4.32) pg/ml respectively. Moreover, a positive association was found between smoking with both TD and FD levels. Higher serum levels of 25-OHD were also found by^41^ in smokers at the time of study than in never smoked and the differences remains unchanged even after correction for the confounders age, sex, physical activity, vitamin D supplementation, season of blood sampling and BMI. Accordingly, in another previous study done in America on 805 women aged between 18 and 33 years, they found that smokers had higher level of 25-OHD than nonsmokers^42^. The above studies confirmed our finding with an exception which is different assessment method was used in each study.

It was suggested that smoking may be less indicator of serum 25-OHD level. However, smoking has become more regulated in Iraq, specifically in our region. Persons who want to smoke usually go outside of the building. In doing so, their exposure to sunlight increases which in turn leads to an increased production of vitamin D among smokers similar to the finding of^42^. In order to be sure if our results agreed or disagreed with the last mentioned finding, the TD and FD results of smokers and nonsmokers were drawn per period of exposure to sunlight. Unfortunately, the results doesn’t prove that smokers exposed to UV light for a period longer than non smokers, so we are uncertain from the causes behind the high results of smokers. Smoking effect on 25-OHD level depends on the assessment method as it was proved by^39^. On that study was hypothesized that the differences in 25-OHD levels when it was measured using 5 different assessment methods could appear due to the presence of interfering substances found in smokers leading to bias the results.

Several studies reported lower serum 25-OHD levels in smokers^43,44^, while others find no significant differences in serum 25-OHD levels between smokers and non-smokers^45,46^.

The body covering varies with women who wear a Hijab (can be colorful), from a loose robe to various types of modern clothing, with skin covering minimum to the wrist, including legs. It allows for the entire face to be revealed, while continuing to cover the hair^47^.

Clothes are a main blocker to sun exposure and therefore 25(OH)D production in skin. Females with western style wearing have higher levels of 25(OH)D than those wearing hijab. Sun exposure to uncovered face and hands as in hijab dressed females is not enough for vitamin D synthesis as it was found by^29^.

A strong correlation between the levels of 25(OH)D and clothing was reported by Mallah and his coworkers in Jordanian women^48^. Also, a lower serum 25(OH)D level was measured in Tunisia with lower mean level of veiled compared to non-veiled women was indicated^49^. The prevalence of vitamin D deficiency was higher in the covered than the uncovered females’ students at Istanbul Medipol University and that vitamin D level is associated with clothing style and age at which the females started wearing hijab as it was proved by^50^.

Regarding the results of this study, firstly, the prevalence of severe deficiency, deficiency, insufficiency and sufficient vitamin D level was higher in the covered group than the uncovered women were detected, it was expected result since the number of the covered women were doubled the uncovered women involved in this study. Secondly, the mean serum level of TD in the veiled women was non-significantly higher than the unveiled women with in the same group which was unexpected result and inconsistent with all of the above-mentioned studies. The veiled subjects (wearing Hijab) whom participated in our study as mentioned in the text, were very similar to the unveiled according to the evaluated parameters. Regarding the duration of body exposure to sunlight it was mentioned in the Tables 8 and 9. The individuals wearing Hijab would just expose their face and hands to the sunlight, the individuals not wearing Hijab would possibly expose a similar area since the evaluation was done during the winter.

Similarly, a non-significant variation was also observed between three groups of women wearing different dress styles in a study done by^31^ in Jordan which was on the same line of our finding. There are several factors related to the dress style that could interact and alter vitamin D level among the population, such as the fabric of the clothing, its texture and color. As our study was performed in Duhok during the winter, all the subjects were dressing similarly (heavy winter clothes). Because of that, we can consider that there was no seasonal variation neither latitude variation nor significant variation in the UV index.

However, the results of our work were in accordance with those stated by^51^ who demonstrated a non-significant Difference of vitamin D levels among hijab users and non-users.

Regarding Free vitamin D level, as there are no previous studies on the impact of the dress style of females on Free vitamin D level, the current result could be considered as the first one in this respect. There is a significant increase in FD level in the veiled women (9.12 ± 4.64 ng/ml) as compared to unveiled (6.16 ± 3.73 ng/ml). Furthermore, there is a significant positive correlation between FD level and dress style whereas a nonsignificant correlation was found with TD level.

The results of the present work demonstrated that 63% of the subjects had a daily exposure to sunlight of around 30 min, yet they weren’t protected from becoming TD deficient. 30% and 26% of the individuals were deficient and severe deficient in vitamin D, respectively. Also, within a group whom their exposure to sunlight was for 30 min/day, the majority of them constituted (69%, 61% and 58%) within their subgroups had severe deficient, deficient and insufficient vitamin D respectively. Strangely, 33% of the participants in the group of exposure for > 1 h to sunlight, they suffered from severe TD deficiency. As the results of exposure to sunlight were drawn per gender, it clarified that females were more commonly susceptible to severe deficiency even with long period of exposure to UV light.

Regarding FD results versus daily exposure to sunlight, just 6% of the participants had abnormally low results of FD and most of them (14 out of 23) their exposure to sunlight was for 30 min/day and 8 of the rest they exposed for more than 1 h to sunlight. Its worthy to mention here that the above-mentioned differences in TD and FD levels according to the daily exposure to sunlight were statistically non-significant, but there is a negative significant association between daily exposure to sunlight with FD level. That mean a highly exposure, led to a decrease in FD level. This result could be considered as a new result in this respect. Although it is well documented that sunlight-induced vitamin D synthesis in the skin is the major source of vitamin D, the precise impact of habitual sunlight exposure on vitamin D levels remain to be further explored, due to several factors that can affect the efficacy of dermal vitamin D production^52^.

The results shown above agreed with the results of others^53,54^ demonstrated no statistically significant differences between sun exposure and serum vitamin D level. The finding of Binkley and his colleagues was very similar to ours, they reported that individuals have different responsiveness to UVB radiation, causing some to have low vitamin D levels despite abundant sun exposure^55^. However, our findings contradicted several previous studies in Southern and Northern Europe as well as other regions of the world, which reported a significant effect of sunlight exposure on 25(OH)D levels^56,57^. It can be concluded in agreement with^55^, that there are factors not yet well understood which can restrict skin production of vitamin D in response to UV radiation and we have to accept the concept that vitamin D deficiency is not due exclusively to inadequate UV exposure.

The true importance of free form of vitamin D has yet to be established through clinical studies mainly pregnancy, fertility, renal and liver diseases^58^*.* Since a number of clinical conditions alter the correlation between TD and FD, a question was raised previously whether the assessment of vitamin D levels might be improved by measuring Free D level instead of or together with TD level^16^. Similar question was indeed our motive to find the correlation between TD and FD but in apparently healthy individuals not clinical conditions in the current study. Up to our knowledge, the above question was not answered previously and the present data could be helpful and the first one in this respect.

Now, after passing through all the studied factors that can affects the levels of TD and FD, it can be concluded that always all the factors had similar impact on both parameters. They were increased with BMI, males had higher mean values of TD and FD than females, both were increased with ages (elderly had higher TD and FD than younger), smoking increases both, wearing hijab also had similar effect on both parameters in addition to daily exposure to sunlight which also had similar effect on both. Spearman’s correlation coefficient proved that the FD was correlated better that the TD with all the studied factors except the gender, no correlation was found.

## Conclusion

It can be concluded that TD and FD mean values increased with BMI, older ages, and in unveiled women. Smokers had higher levels of T and FD levels but cannot be related to prolong period of exposure to UV light. Males had higher levels than females and the severity of deficiency is more common in females than males. FD was negatively associated with daily exposure to sunlight. FD cannot detect all the deficient individuals in TD and the individuals with sufficient TD may be where deficient in FD. Therefore, measurement of FD level in healthy individuals couldn’t add further indication on the real vitamin D status.
